# *catena*-Poly[zinc(II)-bis­[μ_2_-3-(3-pyrid­yl)­benzoato]-κ^2^ *O*:*N*;κ^2^ *N*:*O*]

**DOI:** 10.1107/S1600536811021404

**Published:** 2011-06-11

**Authors:** Long Tang, Ya-Pan Wu, Feng Fu, Xiang-Yang Hou, Qing-Bo Wei

**Affiliations:** a Department of Chemistry and Chemical Engineering, Shaanxi Key Laboratory of Chemical Reaction Engineering, Yan’an University, Yan’an, Shaanxi 716000, People’s Republic of China

## Abstract

In the title compound, [Zn(C_12_H_8_NO_2_)_2_]_*n*_, the Zn^2+^ cation is coordinated by a pair of carboxyl­ate O atoms as well as two pyridyl N atoms to afford a distorted tetra­hedral environment. Adjacent Zn^2+^ cations, with a separation of 8.807 (2) Å, are linked by two 3-(3-pyrid­yl)benzoate ligand bridges, generating an infinite ribbon extending parallel to [001].

## Related literature

For the use of 3-(pyridin-3-yl)benzoate units in the construction of framework structures, see: Guo (2009 ▶). For a similar structure, see: Zhong *et al.* (2008 ▶). 
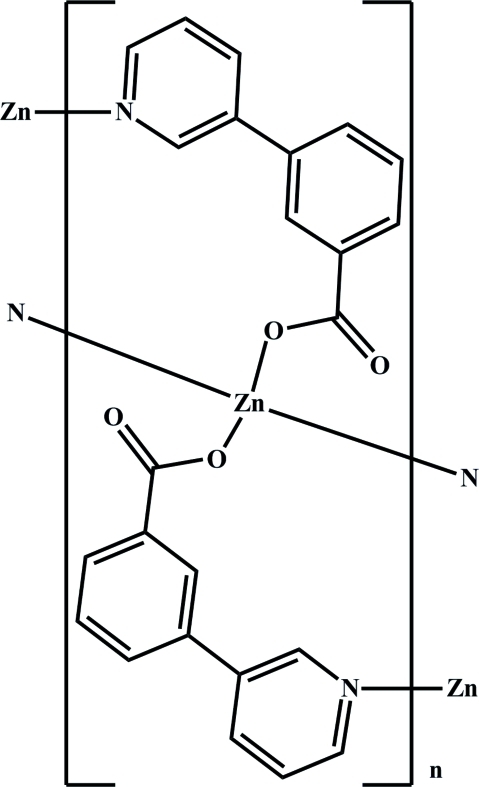


## Experimental

### 

#### Crystal data

[Zn(C_12_H_8_NO_2_)_2_]
*M* *_r_* = 461.76
Monoclinic, 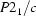

*a* = 10.0512 (8) Å
*b* = 12.0809 (10) Å
*c* = 17.4872 (14) Å
β = 105.631 (1)°
*V* = 2044.9 (3) Å^3^
*Z* = 4
Mo *K*α radiation
μ = 1.24 mm^−1^
*T* = 273 K
0.15 × 0.10 × 0.08 mm

#### Data collection

Bruker SMART CCD diffractometer
Absorption correction: multi-scan (*SADABS*; Sheldrick, 1996 ▶) *T*_min_ = 0.836, *T*_max_ = 0.908
10620 measured reflections
3616 independent reflections
2253 reflections with *I* > 2σ(*I*)
*R*_int_ = 0.050

#### Refinement

*R*[*F* ^2^ > 2σ(*F* ^2^)] = 0.042
*wR*(*F* ^2^) = 0.095
*S* = 1.08
3616 reflections
280 parameters
H-atom parameters constrained
Δρ_max_ = 0.26 e Å^−3^
Δρ_min_ = −0.29 e Å^−3^

### 

Data collection: *SMART* (Bruker, 1997 ▶); cell refinement: *SAINT* (Bruker, 1997 ▶); data reduction: *SAINT*; program(s) used to solve structure: *SHELXS97* (Sheldrick, 2008 ▶); program(s) used to refine structure: *SHELXL97* (Sheldrick, 2008 ▶); molecular graphics: *SHELXTL* (Sheldrick, 2008 ▶); software used to prepare material for publication: *SHELXTL*.

## Supplementary Material

Crystal structure: contains datablock(s) I, global. DOI: 10.1107/S1600536811021404/ng5177sup1.cif

Structure factors: contains datablock(s) I. DOI: 10.1107/S1600536811021404/ng5177Isup2.hkl

Additional supplementary materials:  crystallographic information; 3D view; checkCIF report

## Figures and Tables

**Fig. 1.**
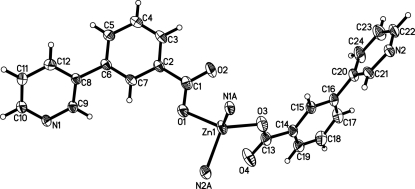
Thermal ellipsoid plot of the title compound at the 30% probability level, hydrogen atoms are drawn as sphere of arbitrary radius.

**Fig. 2.**
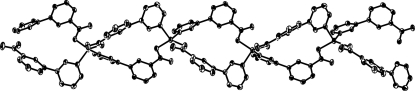
The 1D chain of the title compound, viewed down the c axis.

## supplementary crystallographic information

### Comment

In the structure of the title compound, the Zn^2+^ center is located at the general site and coordinated by a pair of carboxylate oxygen atoms as well as two pyridyl nitrogen donors to afford a tetrahedral environment (see Fig. 1). As a result, the Zn^2+^ ions are connected by the 3-(pyridin-3-yl)benzoate spacers to result in a in?nite 1D double-strand chain motif, with the Zn···Zn separation of 8.807Å, as shown in Fig. 2.

### Experimental

The title compound was prepared by hydrothermal method. An aqueous solution (20 mL) containing 3-(pyridin-3-yl)benzoate acid (0.10 mmol) and Zinc nitrate hexahydrate (0.10 mmol) was placed in a Parr Te?on-lined stainless steel vessel (25 mL) under autogenous pressure, which was heated to 433 K for 72 h and subsequently cooled to room temperature at a rate of 5 K an hour. Colorless single crystals were obtained from the reaction mixture (yield ca 46% based on Zn).

### Refinement

The C-bound H atoms were geometrically placed (C—H = 0.93 Å) and refined as riding with *U*_iso_(H) =1.2*U*_eq_(C).

### Crystal data

**Table:**

[Zn(C_12_H_8_NO_2_)_2_]	*F*(000) = 944
*M**_r_* = 461.76	*D*_x_ = 1.500 Mg m^−^^3^
Monoclinic, *P*2_1_/*c*	Mo *K*α radiation, λ = 0.71073 Å
*a* = 10.0512 (8) Å	Cell parameters from 1527 reflections
*b* = 12.0809 (10) Å	θ = 2.4–21.1°
*c* = 17.4872 (14) Å	µ = 1.24 mm^−^^1^
β = 105.631 (1)°	*T* = 273 K
*V* = 2044.9 (3) Å^3^	Block, colourless
*Z* = 4	0.15 × 0.10 × 0.08 mm

### Data collection

**Table:**

Bruker SMART diffractometer	3616 independent reflections
Radiation source: fine-focus sealed tube	2253 reflections with *I* > 2σ(*I*)
graphite	*R*_int_ = 0.050
φ and ω scans	θ_max_ = 25.1°, θ_min_ = 2.1°
Absorption correction: multi-scan (*SADABS*; Sheldrick, 1996)	*h* = −5→11
*T*_min_ = 0.836, *T*_max_ = 0.908	*k* = −14→14
10620 measured reflections	*l* = −20→20

### Refinement

**Table:**

Refinement on *F*^2^	Primary atom site location: structure-invariant direct methods
Least-squares matrix: full	Secondary atom site location: difference Fourier map
*R*[*F*^2^ > 2σ(*F*^2^)] = 0.042	Hydrogen site location: inferred from neighbouring sites
*wR*(*F*^2^) = 0.095	H-atom parameters constrained
*S* = 1.08	*w* = 1/[σ^2^(*F*_o_^2^) + (0.0353*P*)^2^] where *P* = (*F*_o_^2^ + 2*F*_c_^2^)/3
3616 reflections	(Δ/σ)_max_ = 0.001
280 parameters	Δρ_max_ = 0.26 e Å^−^^3^
0 restraints	Δρ_min_ = −0.29 e Å^−^^3^

### Special details

**Table:**

Geometry. All esds (except the esd in the dihedral angle between two l.s. planes) are estimated using the full covariance matrix. The cell esds are taken into account individually in the estimation of esds in distances, angles and torsion angles; correlations between esds in cell parameters are only used when they are defined by crystal symmetry. An approximate (isotropic) treatment of cell esds is used for estimating esds involving l.s. planes.
Refinement. Refinement of F^2^ against ALL reflections. The weighted R-factor wR and goodness of fit S are based on F^2^, conventional R-factors R are based on F, with F set to zero for negative F^2^. The threshold expression of F^2^ > 2sigma(F^2^) is used only for calculating R-factors(gt) etc. and is not relevant to the choice of reflections for refinement. R-factors based on F^2^ are statistically about twice as large as those based on F, and R- factors based on ALL data will be even larger.

### Fractional atomic coordinates and isotropic or equivalent isotropic displacement parameters (Å^2^)

**Table:**

	*x*	*y*	*z*	*U*_iso_*/*U*_eq_	
Zn1	0.54736 (4)	0.79354 (4)	0.82510 (2)	0.04985 (17)	
C1	0.4220 (4)	0.6225 (3)	0.8717 (2)	0.0479 (9)	
C2	0.3496 (3)	0.5541 (3)	0.92062 (18)	0.0401 (9)	
C3	0.2852 (4)	0.4556 (3)	0.8909 (2)	0.0478 (10)	
H3	0.2882	0.4309	0.8410	0.057*	
C4	0.2170 (4)	0.3943 (3)	0.9347 (2)	0.0525 (10)	
H4	0.1750	0.3279	0.9146	0.063*	
C5	0.2105 (4)	0.4308 (3)	1.0087 (2)	0.0473 (9)	
H5	0.1635	0.3893	1.0379	0.057*	
C6	0.2743 (3)	0.5295 (3)	1.03928 (18)	0.0389 (9)	
C7	0.3441 (3)	0.5902 (3)	0.99507 (18)	0.0415 (9)	
H7	0.3879	0.6558	1.0155	0.050*	
C8	0.2645 (4)	0.5697 (3)	1.11789 (18)	0.0379 (8)	
C9	0.3784 (4)	0.6111 (3)	1.17361 (18)	0.0415 (9)	
H9	0.4620	0.6142	1.1604	0.050*	
C10	0.2537 (4)	0.6462 (3)	1.2631 (2)	0.0519 (10)	
H10	0.2495	0.6732	1.3123	0.062*	
C11	0.1359 (4)	0.6076 (3)	1.2119 (2)	0.0556 (11)	
H11	0.0529	0.6080	1.2259	0.067*	
C12	0.1416 (4)	0.5679 (3)	1.1389 (2)	0.0512 (10)	
H12	0.0624	0.5397	1.1037	0.061*	
C13	0.7782 (4)	0.7247 (3)	0.7844 (2)	0.0525 (10)	
C14	0.8517 (4)	0.6655 (3)	0.73188 (19)	0.0444 (9)	
C15	0.7794 (4)	0.6278 (3)	0.65703 (19)	0.0449 (9)	
H15	0.6856	0.6430	0.6380	0.054*	
C16	0.8458 (4)	0.5675 (3)	0.6104 (2)	0.0474 (10)	
C17	0.9861 (4)	0.5446 (3)	0.6400 (2)	0.0607 (11)	
H17	1.0310	0.5021	0.6102	0.073*	
C18	1.0589 (4)	0.5845 (3)	0.7132 (2)	0.0664 (12)	
H18	1.1535	0.5719	0.7317	0.080*	
C19	0.9909 (4)	0.6432 (3)	0.7588 (2)	0.0553 (10)	
H19	1.0400	0.6683	0.8087	0.066*	
C20	0.7694 (4)	0.5294 (3)	0.52977 (19)	0.0475 (9)	
C21	0.6937 (4)	0.6032 (3)	0.47544 (19)	0.0494 (10)	
H21	0.6875	0.6758	0.4918	0.059*	
C22	0.6343 (4)	0.4723 (4)	0.3773 (2)	0.0625 (12)	
H22	0.5881	0.4524	0.3256	0.075*	
C23	0.7069 (5)	0.3932 (4)	0.4277 (3)	0.0826 (15)	
H23	0.7096	0.3207	0.4102	0.099*	
C24	0.7762 (5)	0.4218 (4)	0.5045 (2)	0.0742 (13)	
H24	0.8270	0.3691	0.5390	0.089*	
N1	0.3752 (3)	0.6471 (2)	1.24571 (15)	0.0437 (7)	
N2	0.6278 (3)	0.5766 (3)	0.39999 (16)	0.0497 (8)	
O1	0.4801 (2)	0.7115 (2)	0.90317 (13)	0.0540 (7)	
O2	0.4192 (3)	0.5923 (2)	0.80425 (14)	0.0648 (8)	
O3	0.6515 (3)	0.7467 (2)	0.75352 (14)	0.0635 (8)	
O4	0.8408 (3)	0.7481 (3)	0.85317 (16)	0.0880 (10)	

### Atomic displacement parameters (Å^2^)

**Table:**

	*U*^11^	*U*^22^	*U*^33^	*U*^12^	*U*^13^	*U*^23^
Zn1	0.0573 (3)	0.0665 (3)	0.0242 (2)	0.0047 (2)	0.00805 (19)	0.0020 (2)
C1	0.050 (2)	0.059 (3)	0.031 (2)	0.013 (2)	0.0045 (18)	0.0057 (19)
C2	0.043 (2)	0.047 (2)	0.0264 (18)	0.0099 (18)	0.0020 (16)	0.0005 (16)
C3	0.054 (3)	0.052 (3)	0.032 (2)	0.013 (2)	0.0025 (18)	−0.0080 (18)
C4	0.057 (3)	0.051 (3)	0.044 (2)	0.000 (2)	0.004 (2)	−0.0111 (19)
C5	0.047 (2)	0.053 (3)	0.039 (2)	−0.0027 (19)	0.0055 (17)	−0.0026 (18)
C6	0.038 (2)	0.046 (2)	0.0277 (19)	0.0053 (18)	0.0002 (16)	−0.0003 (16)
C7	0.046 (2)	0.045 (2)	0.0298 (19)	0.0021 (17)	0.0026 (17)	−0.0033 (16)
C8	0.040 (2)	0.044 (2)	0.0280 (18)	0.0017 (17)	0.0063 (16)	0.0005 (15)
C9	0.041 (2)	0.052 (2)	0.031 (2)	0.0046 (18)	0.0090 (16)	0.0023 (16)
C10	0.062 (3)	0.057 (3)	0.040 (2)	−0.008 (2)	0.021 (2)	−0.0050 (19)
C11	0.053 (3)	0.067 (3)	0.055 (3)	−0.008 (2)	0.028 (2)	−0.013 (2)
C12	0.049 (3)	0.056 (3)	0.047 (2)	−0.008 (2)	0.0096 (19)	−0.0052 (19)
C13	0.068 (3)	0.056 (3)	0.033 (2)	0.005 (2)	0.014 (2)	0.0092 (18)
C14	0.051 (3)	0.052 (2)	0.0290 (19)	0.0014 (19)	0.0089 (18)	0.0098 (16)
C15	0.049 (2)	0.052 (2)	0.031 (2)	0.0046 (19)	0.0079 (17)	0.0097 (17)
C16	0.059 (3)	0.052 (2)	0.034 (2)	0.004 (2)	0.0172 (19)	0.0094 (17)
C17	0.062 (3)	0.080 (3)	0.043 (2)	0.019 (2)	0.018 (2)	0.005 (2)
C18	0.051 (3)	0.095 (4)	0.053 (3)	0.012 (2)	0.013 (2)	0.010 (2)
C19	0.058 (3)	0.070 (3)	0.035 (2)	−0.003 (2)	0.007 (2)	0.005 (2)
C20	0.064 (3)	0.047 (3)	0.033 (2)	0.005 (2)	0.0150 (18)	0.0004 (18)
C21	0.064 (3)	0.050 (2)	0.032 (2)	−0.003 (2)	0.0087 (18)	−0.0055 (17)
C22	0.094 (3)	0.062 (3)	0.035 (2)	−0.015 (3)	0.024 (2)	−0.011 (2)
C23	0.142 (5)	0.050 (3)	0.057 (3)	−0.003 (3)	0.029 (3)	−0.007 (2)
C24	0.123 (4)	0.058 (3)	0.044 (3)	0.018 (3)	0.026 (3)	0.010 (2)
N1	0.049 (2)	0.055 (2)	0.0286 (16)	−0.0032 (15)	0.0128 (14)	−0.0033 (14)
N2	0.061 (2)	0.056 (2)	0.0307 (17)	−0.0053 (16)	0.0102 (15)	−0.0023 (15)
O1	0.0694 (18)	0.0601 (18)	0.0311 (13)	−0.0095 (15)	0.0109 (12)	−0.0004 (12)
O2	0.088 (2)	0.078 (2)	0.0307 (15)	0.0024 (15)	0.0201 (14)	−0.0066 (13)
O3	0.0587 (19)	0.092 (2)	0.0411 (16)	0.0112 (16)	0.0162 (14)	−0.0029 (14)
O4	0.100 (2)	0.121 (3)	0.0370 (17)	0.028 (2)	0.0069 (16)	−0.0116 (17)

### Geometric parameters (Å, °)

**Table:**

Zn1—O3	1.921 (2)	C12—H12	0.9300
Zn1—O1	1.949 (2)	C13—O4	1.230 (4)
Zn1—N1^i^	2.035 (3)	C13—O3	1.270 (4)
Zn1—N2^ii^	2.064 (3)	C13—C14	1.506 (5)
C1—O2	1.227 (4)	C14—C19	1.377 (5)
C1—O1	1.276 (4)	C14—C15	1.392 (4)
C1—C2	1.510 (5)	C15—C16	1.391 (5)
C2—C3	1.387 (5)	C15—H15	0.9300
C2—C7	1.388 (4)	C16—C17	1.393 (5)
C3—C4	1.374 (5)	C16—C20	1.485 (5)
C3—H3	0.9300	C17—C18	1.380 (5)
C4—C5	1.385 (4)	C17—H17	0.9300
C4—H4	0.9300	C18—C19	1.379 (5)
C5—C6	1.391 (4)	C18—H18	0.9300
C5—H5	0.9300	C19—H19	0.9300
C6—C7	1.385 (4)	C20—C21	1.373 (5)
C6—C8	1.486 (4)	C20—C24	1.380 (5)
C7—H7	0.9300	C21—N2	1.346 (4)
C8—C12	1.380 (4)	C21—H21	0.9300
C8—C9	1.382 (4)	C22—N2	1.327 (4)
C9—N1	1.343 (4)	C22—C23	1.370 (5)
C9—H9	0.9300	C22—H22	0.9300
C10—N1	1.335 (4)	C23—C24	1.381 (5)
C10—C11	1.361 (5)	C23—H23	0.9300
C10—H10	0.9300	C24—H24	0.9300
C11—C12	1.380 (5)	N1—Zn1^ii^	2.035 (3)
C11—H11	0.9300	N2—Zn1^i^	2.064 (3)
			
O3—Zn1—O1	131.25 (11)	O4—C13—C14	120.0 (4)
O3—Zn1—N1^i^	99.90 (11)	O3—C13—C14	116.1 (3)
O1—Zn1—N1^i^	105.38 (11)	C19—C14—C15	118.8 (3)
O3—Zn1—N2^ii^	116.65 (12)	C19—C14—C13	120.3 (3)
O1—Zn1—N2^ii^	95.30 (11)	C15—C14—C13	120.8 (3)
N1^i^—Zn1—N2^ii^	106.30 (12)	C16—C15—C14	120.8 (3)
O2—C1—O1	123.7 (4)	C16—C15—H15	119.6
O2—C1—C2	119.4 (4)	C14—C15—H15	119.6
O1—C1—C2	116.8 (3)	C15—C16—C17	118.9 (3)
C3—C2—C7	119.3 (3)	C15—C16—C20	120.8 (3)
C3—C2—C1	120.3 (3)	C17—C16—C20	120.3 (3)
C7—C2—C1	120.4 (3)	C18—C17—C16	120.4 (4)
C4—C3—C2	120.4 (3)	C18—C17—H17	119.8
C4—C3—H3	119.8	C16—C17—H17	119.8
C2—C3—H3	119.8	C17—C18—C19	119.7 (4)
C3—C4—C5	120.4 (4)	C17—C18—H18	120.2
C3—C4—H4	119.8	C19—C18—H18	120.2
C5—C4—H4	119.8	C14—C19—C18	121.3 (4)
C4—C5—C6	119.9 (4)	C14—C19—H19	119.4
C4—C5—H5	120.0	C18—C19—H19	119.4
C6—C5—H5	120.0	C21—C20—C24	117.1 (3)
C7—C6—C5	119.3 (3)	C21—C20—C16	120.3 (3)
C7—C6—C8	120.9 (3)	C24—C20—C16	122.5 (3)
C5—C6—C8	119.8 (3)	N2—C21—C20	123.9 (3)
C6—C7—C2	120.7 (3)	N2—C21—H21	118.0
C6—C7—H7	119.6	C20—C21—H21	118.0
C2—C7—H7	119.6	N2—C22—C23	122.1 (4)
C12—C8—C9	116.7 (3)	N2—C22—H22	119.0
C12—C8—C6	121.9 (3)	C23—C22—H22	119.0
C9—C8—C6	121.4 (3)	C22—C23—C24	119.5 (4)
N1—C9—C8	123.6 (3)	C22—C23—H23	120.3
N1—C9—H9	118.2	C24—C23—H23	120.3
C8—C9—H9	118.2	C20—C24—C23	119.5 (4)
N1—C10—C11	122.7 (3)	C20—C24—H24	120.3
N1—C10—H10	118.7	C23—C24—H24	120.3
C11—C10—H10	118.7	C10—N1—C9	117.8 (3)
C10—C11—C12	118.8 (4)	C10—N1—Zn1^ii^	120.6 (2)
C10—C11—H11	120.6	C9—N1—Zn1^ii^	121.5 (2)
C12—C11—H11	120.6	C22—N2—C21	117.9 (3)
C11—C12—C8	120.3 (3)	C22—N2—Zn1^i^	125.1 (3)
C11—C12—H12	119.9	C21—N2—Zn1^i^	116.5 (3)
C8—C12—H12	119.9	C1—O1—Zn1	109.3 (2)
O4—C13—O3	123.9 (4)	C13—O3—Zn1	116.4 (2)

Symmetry codes: (i) *x*, −*y*+3/2, *z*−1/2; (ii) *x*, −*y*+3/2, *z*+1/2.
